# Altered expression of circular RNA in patients with cervical artery dissection

**DOI:** 10.3389/fneur.2023.1228400

**Published:** 2023-10-16

**Authors:** Yifan Wang, Zhaofei Dong, Jie Li, Yudi Li, Jianyi Mai, Wenru Tan, Siqi Yang, Li Ling, Yajie Liu

**Affiliations:** ^1^Department of Neurology, Shenzhen Hospital, Southern Medical University, Shenzhen, Guangdong, China; ^2^Departments of Neurology, The Eighth Affiliated Hospital, Sun Yat-sen University, Shenzhen, Guangdong, China

**Keywords:** cervical artery dissection, circRNA, ischemic stroke, high-throughput sequencing, biomark

## Abstract

Cervical artery dissection (CeAD), a special cerebrovascular disease and the main cause of stroke in young people, can present with ischemic stroke, headache, subarachnoid hemorrhage, and other symptoms, increasing the possibility of misdiagnosis. As a special class of non-coding RNAs, circRNAs are commonly found in organisms and can play regulatory roles in transcription and post-transcription processes, affecting gene expression.CircRNAs have reported to be associated with neurological diseases; however, their role in CeAD has not been discerned. In this study, we aimed to elucidate the pathophysiological changes in patients with CeAD and identify biomarkers. Peripheral blood mononuclear cells from patients with CeAD and healthy controls were sequenced using high-throughput sequencing. We detected 460 differently expressed circRNAs in patients with CeAD (*p* < 0.5, fold difference ≥ 2), of which 240 were upregulated and 220 were downregulated. Four circRNAs showed significant differences in expression, which were validated using qRT-PCR. These results suggested that three circRNAs were consistent with high-throughput sequencing results. Bioinformatics analysis demonstrated that these differentially expressed circRNAs were involved in protein metabolism, regulation, synapses, and other pathophysiological processes during CeAD-induced stroke. Additionally, various pathways related to inflammation were closely associated with circRNAs. Based on our results, we suggest that the aberrant expression of circRNAs in CeAD may serve as a biomarker for its diagnosis and as a potential therapeutic target.

## 1. Introduction

Cervical artery dissection (CeAD) is a major cause of stroke in young individuals, with CeAD causing 2% of ischemic strokes, and the rate in stroke patients less than 45 years of age being as high as 8–25% (1–3). Arterial dissection is the formation of an intramural hematoma within the wall of an arterial intimal dissection that causes blood to flow into the canal. Current studies suggest that intimal dissection and sudden rupture of the internal elastic lamina are the main etiologies (4, 5). Irreversible damage to the intima and internal elastic plate may be the pathological basis for CeAD formation (6). However, studies on the pathophysiological aspects of CeAD are scarce. Nevertheless, Previous research demonstrated that cerebrovascular disease risk factors such as hypertension, hyperhomocysteinemia, atherosclerosis, and hereditary connective tissue diseases such as Marfan syndrome are closely related to it, and some spontaneous dissections are considered to be caused by structural abnormalities of the vessel wall (7–9). Studies have also suggested that infections occurring in the short term are risk factors for spontaneous dissections (10).

With increasing RNA research, it has been found that non-coding RNAs are widely involved in various pathophysiological processes, such as epigenetic, transcriptional, and post-transcriptional regulation, and are closely related to many diseases (11). Compared to the conventional inclusion of linear RNA with 3′ and 5′ ends that exhibits a closed loop structure, circRNAs, a special class of non-coding RNAs, are less susceptible to exonucleases, and has more stable expression, thereby being a potential biomarker (12). Recent studies have confirmed that circRNAs are widely expressed in the brain tissues of mammals, rodents, and humans (13). Dong et al. found that circRNAs not only exist but also influence the pathophysiology of Actue ischemic stroke in cerebrovascular diseases (14). Moreover, some scholars applied the glucose oxygen deprivation/reoxygenation model to HT22 cells and found that 15 circRNAs were altered in the model group, among which mmu-circRNA-015947 might participate in the process of cerebral ischemia–reperfusion injury (15). Decreased circRNA-ciRS7 expression has been observed in the hippocampal tissue of Alzheimer’s disease patients, and the loss of ciRS7 causes an increase in miRNA-7 expression through sponge adsorption and downregulates the expression of target proteins of miRNA-7, thus leading to a reduction in clearance (16). Additionally, numerous studies have confirmed the close association of circRNAs with neurological diseases; however, until now, there has been no research on circRNAs in CeAD.

The diagnosis of CeAD depends mainly on imaging and clinical manifestations. Cerebrovascular angiography is the gold standard for the diagnosis of CeAD. However, it is a complex examination method, which makes rapid diagnosis difficult. Based on the existing research results on circRNAs in the nervous system, this study used high-throughput sequencing to conduct a prospective controlled study on patients with CeAD and healthy subjects, to clarify the pathophysiological changes in patients with CeAD from a new perspective, and to predict diagnostic markers and future therapeutic targets.

## 2. Materials and methods

### 2.1. Patient samples

We included 10 CeAD patients and 10 normal controls in this study. All CeAD patients included in this study were patients at the Department of Neurology, ShenZhen Hospital of Southern Medical University, from 2020 to 2022, while the healthy controls were volunteers from the Health Management Center at Shenzhen Hospital of Southern Medical University. The study was approved by the Ethics Committee of the Shenzhen Hospital of Southern Medical University (NYSZYYEC20200016), and all samples for the trial were obtained from the patients and controls upon the receipt of signed informed consent forms.

### 2.2. Diagnostic criteria

#### 2.2.1. Diagnostic criteria for ischemic stroke

All patients were diagnosed using a Computed Tomography (CT) or Magnetic Resonance Imaging(MRI) scan, according to the American Heart Association/American Stroke Association diagnostic criteria for cerebral infarction (17).

#### 2.2.2. Diagnostic criteria for CeAD

1.Clinical manifestations included stroke event, neurological deficits, headache and neck pain, and Horner’s syndrome.

2.Imaging: Ultrasound of the neck indicates double-lumen structure in the blood vessel; CT angiography or magnetic resonance angiography indicates stenosis, occlusion, pseudoaneurysm, intimal flap, and the double-lumen; digital subtraction angiography shows stenosis, occlusion, or the “flame sign,” “rat tail sign,” and “thread-like sign.” For digital subtraction angiography, stenosis, occlusion, “flame sign,” “rat tail sign,” “thread-like sign,” atherosclerosis, and embolism were excluded in all patients.

3.CeAD with rheumatic diseases and connective tissue diseases should also be excluded.

#### 2.2.3. Peripheral blood mononuclear cell (PBMC) separation

Venous blood samples (10 mL) were collected from patients with CeAD, and PBMCs were extracted as previously described (18).

### 2.3. RNA extraction and high-throughput sequencing of RNA samples

Total RNA was isolated from PBMCs using the Magzol Reagent (Magen, China), as per manufacturer’s protocol. The quantity and integrity of the RNA was assessed using the K5500 micro-spectrophotometer (Beijing Kaiao, China) and the Agilent 2,200 TapeStation (Agilent Technologies, United States), respectively. Briefly, rRNAs were removed from the total RNA using the QIAseq FastSelect-rRNA HMR Kit (QIAGEN, Germany). The RNA was then treated with RNase R (Epicenter, United States) and fragmented to approximately 200 bp. Subsequently, the purified RNA fragments were subjected to first strand and second strand cDNA synthesis, followed by adaptor ligation and enrichment with a low cycle according to manufacturer’s instructions for the NEBNext® Ultra™ RNA Library Prep Kit for Illumina (NEB, United States). The library products were evaluated using the Agilent 2,200 TapeStation and Qubit (Thermo Fisher Scientific, United States), and further sequenced using an Illumina (Illumina, United States), through the paired-end 150 bp approach, at Ribobio Co. Ltd. (Ribobio, China).

### 2.4. Identification and quantification of circRNAs

The circRNAs were detected using the CIRI2 and CIRCexplorer2 algorithms. Reads were mapped to the human reference genome grch37/hg19 using the BWA-MEM or TopHat tools, respectively.^1^ CIRI2 detected pairwise staggered splice reporter signals from the read mapping information through local alignment with BWA-MEM and incorporated systematic filtering steps to remove potential false positives. CIRCexplorer2 was exported to detect circRNAs using TopHat and TopHat fusion alignments. If a circRNA was detected using both the methods, it was considered to be recognized. The DESeq2 package in Bioconductor was used to identify differentially expressed circRNAs according to the criteria of |log_2_ (fold change) | > 1 and *p* ≤ 0.05.

### 2.5. Validation of high-throughput sequencing using qRT-PCR

To ensure the validity of the high-throughput sequencing results, the experiments were validated using qRT-PCR with β-actin as an internal control, and the samples were prepared in triplicates. Total RNA was reverse-transcribed to complementary DNA using a reverse transcription kit (Takara Bio). We performed qRT-PCR to detect circRNA expression levels using the SYBR Green qPCR SuperMix and the 7,500 Sequence Detection System (Applied Biosystems). The primers used are listed in (Table 1). Ploidy changes in all circRNA expression profiles were detected via the 2-ΔΔCt method.

**Table 1:** Primers used in validation experiments.

circRNAs	Primer sequence (5′-3′)	Product length (bp)
Has_circ:chr17:81042814–81,043,199	F: GACCACAGGCTTCCAGTACGA R: CACAGCGCAGATACACCTGCT	148
Has_circ:chr1:2234417-2236024(h-has-circ-0007120)	F: AGTTCCTGCATGAGGTGGTCAA R: CGGCTTGTCCTTTTCGGAAG	173
Has_circ:chr1:31465237-31479949(h-has-circ-0000044)	F: GGCATGGAGCCTCTTCAGTTT R: TCCATCTTTGCTGGATTCATCTGT	153
Has_circ:chr14:35269430–35,272,194	F: GCAGATGCCCTAGAAGCGA R: GCAGATGCCCTAGAAGCGA	203

bp, base pair; circRNA, Circular RNA; F, forward; R, reverse.

### 2.6. Data analysis

#### 2.6.1. Go terms and KEGG pathway enrichment analysis

GO functional annotations and KEGG pathway enrichment analyses were performed using the clusterProfiler package in the Bioconductor/KOBAS 3.0. For KEGG enrichment analysis, a *p* < 0.05 was used as the threshold to determine significant enrichment of the gene sets.

#### 2.6.2. Analysis of circRNA and miRNA interaction

Acting as an miRNA sponge, circRNA inhibits the negative regulation of its target mRNA by competitively binding to miRNA with mRNA; therefore, the prediction of miRNA that can be bound by circRNA can effectively predict the potential function of circRNA. Further functional annotation of the three circRNAs identified by qRT-PCR was performed. We used the miRanda, RNAhybrid, and TargetScan tools to predict circRNA and its miRNA binding site and obtained the prediction results from the intersection of each tool.

### 2.7. Statistical analysis

In this study, all data are presented as mean ± standard deviation, and the difference between groups was determined using the t-test, with *p* < 0.05 identified as a statistically significant difference.

## 3. Results

### 3.1. Data analysis

**Figure 1:**
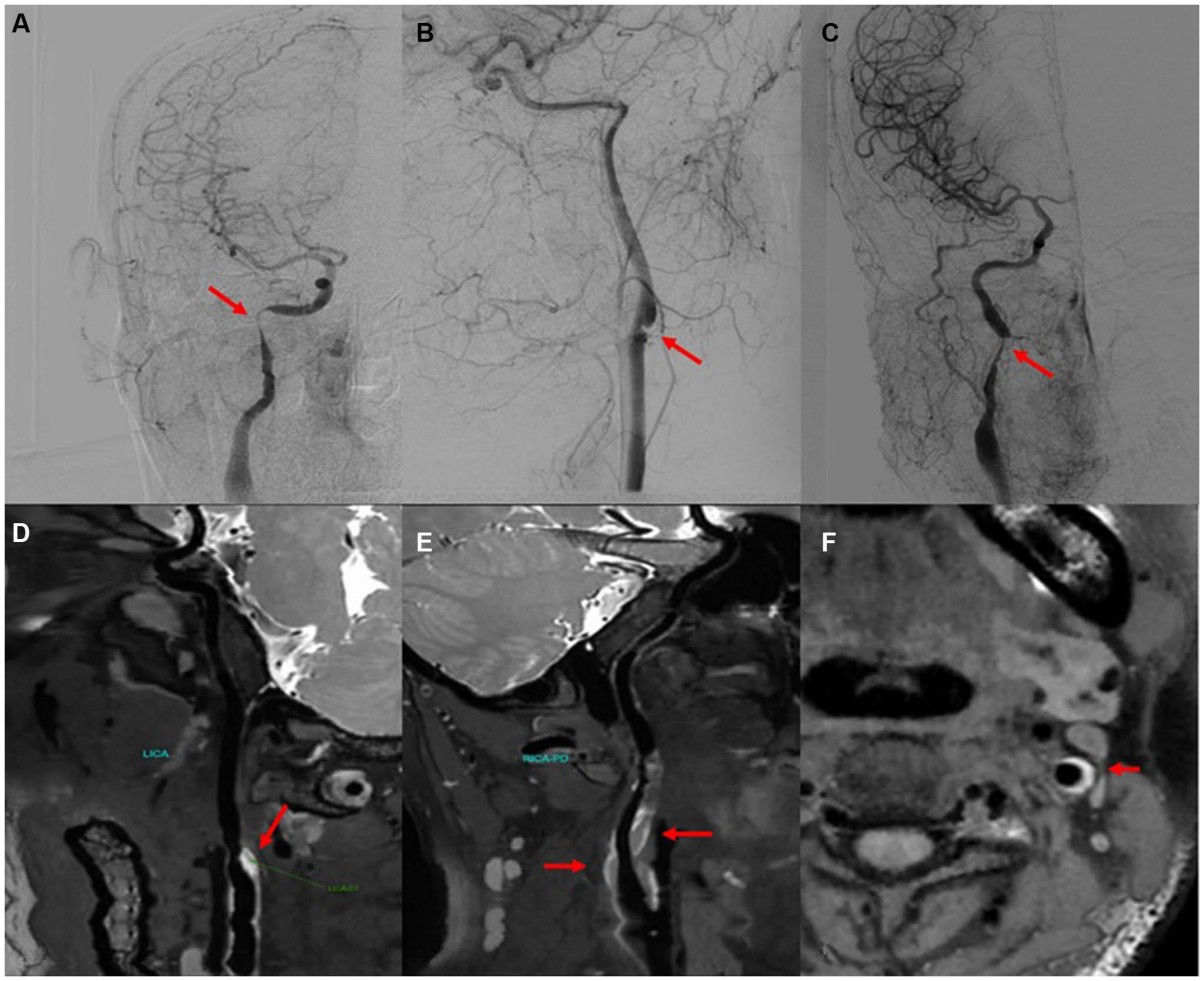
CeAD imaging (arrow). **(A–C)** Digital subtraction angiography showing vascular dissection. **(D,E)** High resolution magnetic resonance demonstrating vascular lumen wall hematoma (white high signal). **(F)** Axial MRI scan showing a mural hematoma and residual lumen.

We included 10 patients diagnosed with CeAD and 10 healthy controls in this trial; The clinical symptoms of CeAD patients include stroke events, neurological deficits, head and neck pain, etc. The average age of CeAD patients was 40.67 ± 7.97 years (age range: 32–55), and a mean NIHSS score of 2.3 (ranging from 1 to 12 points). Among CeAD patients, 5 were caused by minor trauma, including 4 patients who developed clinical symptoms after head and neck massage, and another patient who injured the neck while playing basketball. Clinical characteristics of CeAD patients are detailed in Tables 2, 3. The imaging presentation is shown in Figure 1.

**Table 2:** Characteristics of the study population.

	CeAD (*n* = 10)	Control (*n* = 10)	*p*-value
Age (years)	40.67 ± 7.97	28.4 ± 8.07	*p* = 0.035
Sex			
Male	6	5	NA
Female	4	5	NA
NIHSS score	2.3 ± 3.65	0	NA
Hypertension	2	1	NA
Diabetes mellitus	0	0	NA
Smoking	5	3	NA
Hyperlipemia	2	0	
White blood cell	10.25 ± 2.59	5.99 ± 1.19	*p*<0.001
Neutrophile granulocyte	7.21 ± 2.06	3.22 ± 0.66	*p*<0.001

**Table 3:** Baseline characteristics of patients with CeAD.

Number	Age/sex	Presentation	Site of lesion	Etiology	Medicine
1	37/male	Hemiparesis	LICA (C1)	Spontaneous	Antiplatelet
2	48/male	Hemiparesis, aphasia	RICA (origin)	Spontaneous	Antiplatelet
3	46/female	Headache	RICA (origin)	Minor trauma	Antiplatelet
4	55/male	Neck pain	RICA (C1)	Spontaneous	Antiplatelet
5	35/male	Hemiparesis	LICA (C1)	Minor trauma	Antiplatelet
6	40/male	Unconsciousness	LVA (V3)	Minor trauma	Antiplatelet
7	32/female	Hemiparesis, aphasia	LICA (C1)	Spontaneous	Antiplatelet
8	32/male	Vertigo	RVA (V3)	Minor trauma	Antiplatelet
9	35/female	Neck pain,Vertigo	RVA (V3)	Minor trauma	Anticoagulation
10	43/female	Hemiparesis, aphasia	LICA (C1)	Spontaneous	Anticoagulation

RICA, Right internal carotid artery; LICA, Left internal carotid artery; LVA, Left vertebral artery; Minor trauma: (a) only obtained in the history with a potential temporal association to the occurrence of dissection and (b) did not require a visit to the emergency room or doctor’s office.

### 3.2. CircRNA profiles

**Figure 2:**
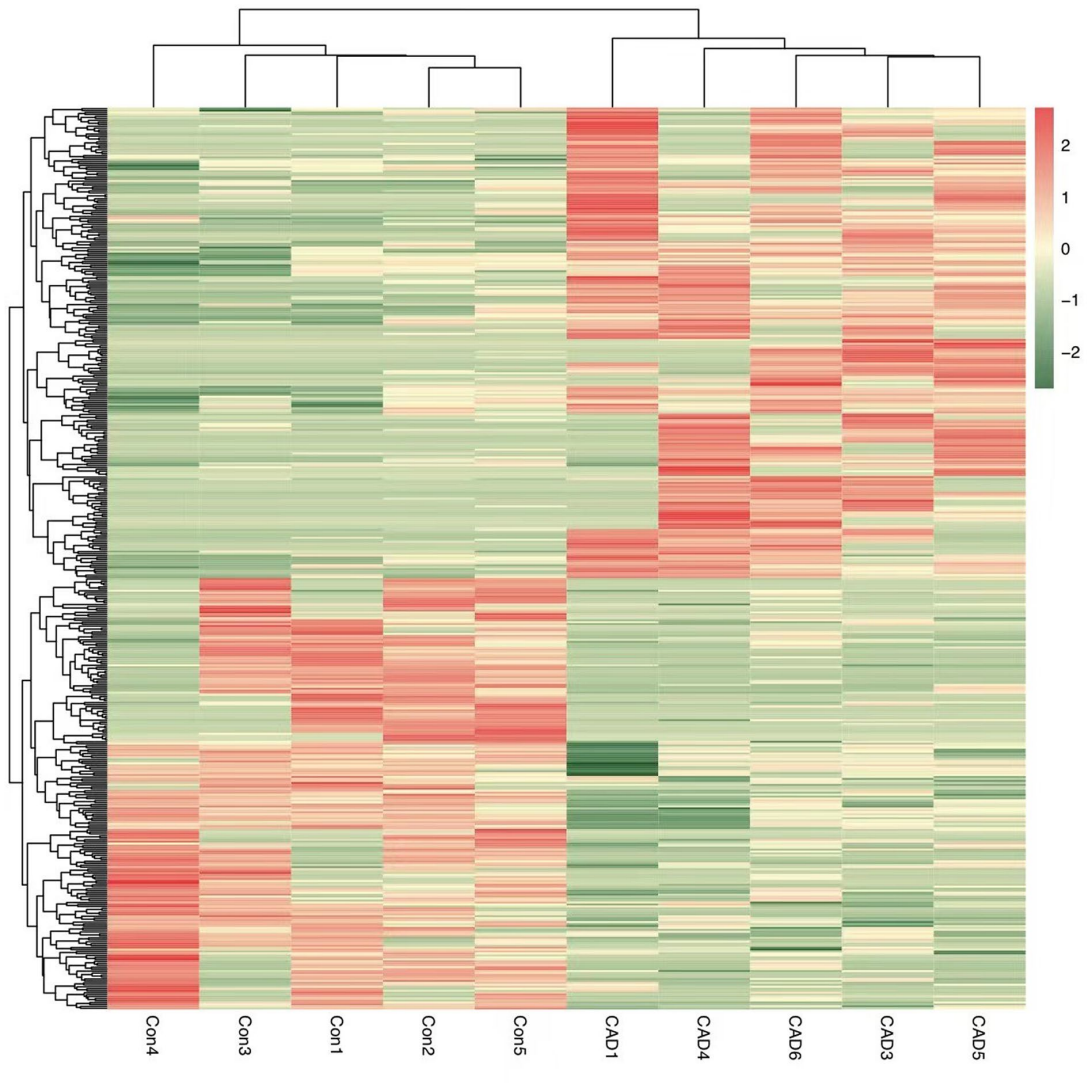
Heat map of circRNAs differentially expressed between the CeAD and control groups. Red represents high expression and green represents low expression.

**Figure 3:**
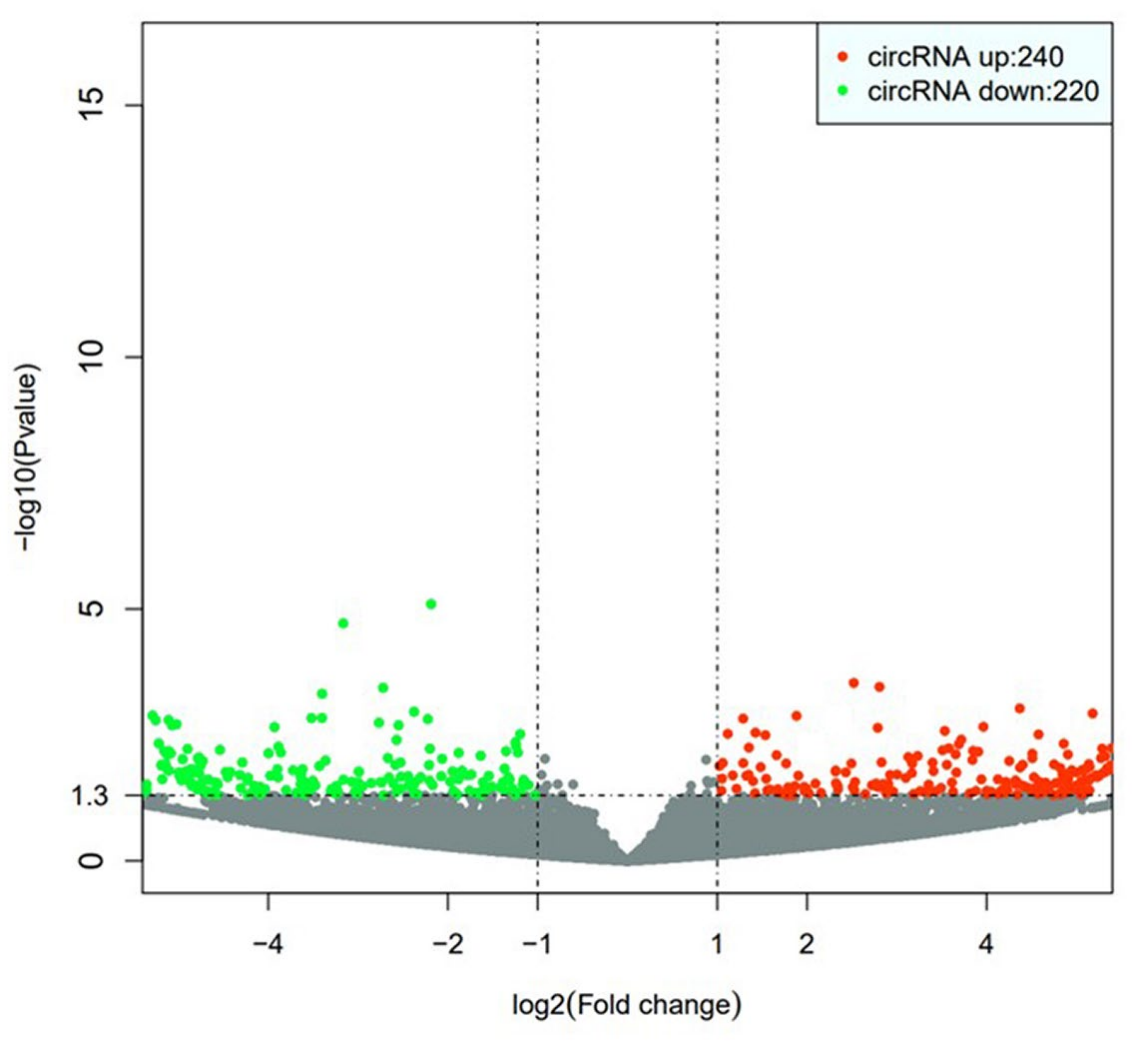
Volcano plot, the horizontal coordinate represents the change of circRNA expression in different samples; the vertical coordinate represents the statistical significance of the difference of circRNA expression change, the red dots indicate the upregulated circRNA, and the green dots indicate the downregulated circRNA.

By analyzing the PBMCs from the patients and controls using high-throughput sequencing, 30,990 circRNAs were obtained. Using the DESeq2 package, 460 differentially expressed circRNAs, including 240 upregulated circRNAs and 220 downregulated circRNAs, were detected. Hierarchical clustering of the aberrantly expressed circRNAs was performed. Volcano plots were used to visualize circRNAs that differed between the CeAD and control group (Figure 2), and scatter plots were used to evaluate the changes in circRNA expression (Figure 3). The circRNAs has_circ:chr17:81042814–81,043,199, has_circ:chr1:2234417-2236024, has_circ:chr1:31465237–31,479,949, has_circ:chr14:35269430–35,272,194 were regulated with the highest fold differences, indicating that these circRNAs are potential biomarkers for CeAD.

### 3.3. Validation using qRT-PCR

**Figure 4:**
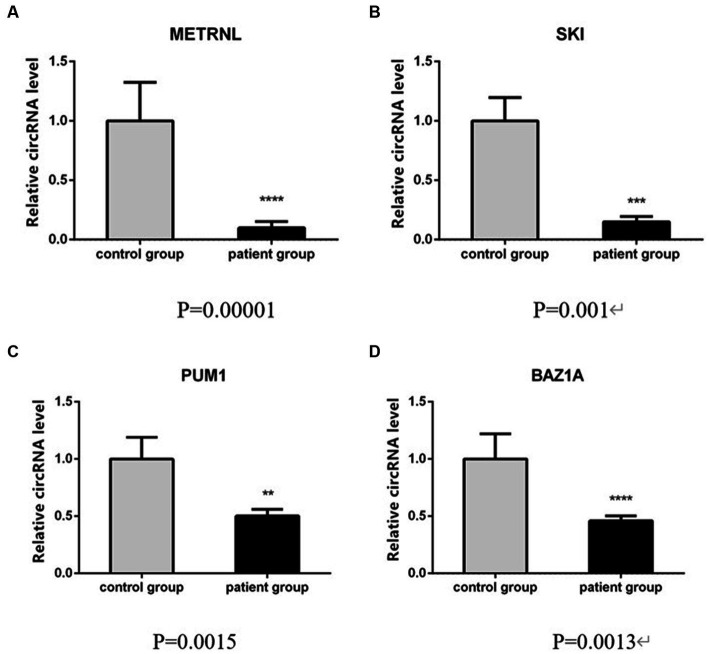
qRT-PCR validation of circRNA differential levels in high-throughput sequencing, (samples in triplicates, values are mean ± SD (*n* = 3 per group), qRT-PCR analysis was performed in triplicates).

To verify the high-throughput sequencing results according to the degree of fold change (fold change ≥2, *p* < 0.05) of the 460 differentially expressed circRNAs in this study, four circRNAs with the greatest difference between the upregulated and downregulated circRNAs (Table 4) were selected and detected using qRT-PCR, in 10 patients with CeAD and 10 controls. The results of the three downregulated groups (has_circ:chr17:81042814–81,043,199, has_circ:chr1:2234417–2,236,024, has_circ:chr1:31465237–31,479,949) were consistent with the high-throughput sequencing results and the differences were statistically significant, whereas the results of the upregulated groups has_circ:chr14:35269430–35,272,194 were inconsistent with the high-throughput sequencing results (Figure 4).

**Table 4:** Information on qRT-PCR Validation Experiments.

CircName	Gene symbol	Log_2_ (Fold_change)	*p*-value
Has_circ:chr17:81042814–81,043,199	METRNL	−3.164406947	0.0000193
Has_circ:chr1:2234417–2,236,024	SKI	−2.375733233	0.001099435
Has_circ:chr1:31465237–31,479,949	PUM1	−2.223565792	0.001537659
Has_circ:chr14:35269430–35,272,194	BAZ1A	1.882103238	0.00132595

### 3.4. Bioinformatics analysis

#### 3.4.1. Go and KEGG analyses

**Figure 5:**
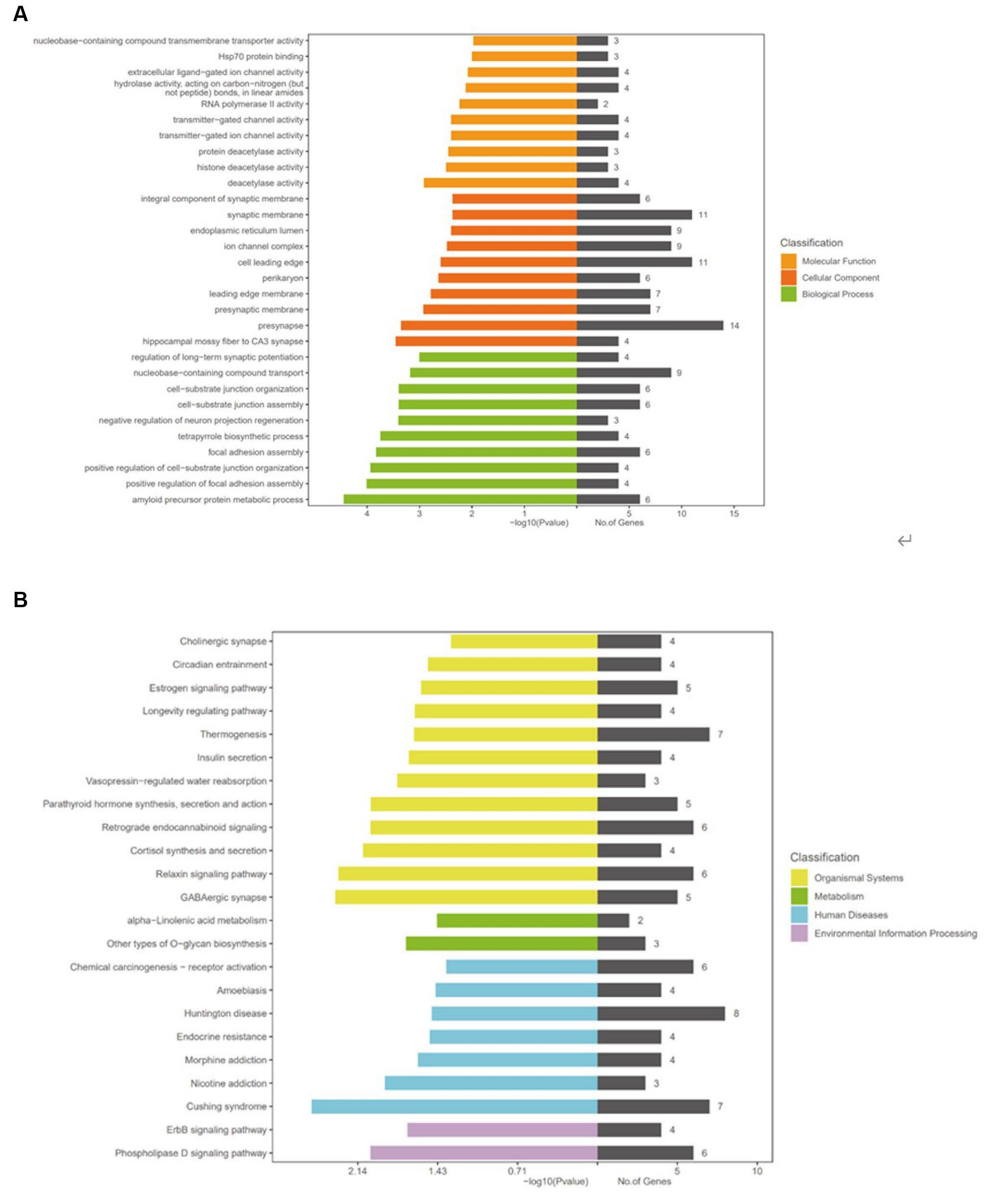
GO and KEGG analyses.

To further study the function and role of circRNAs in CeAD, we performed the KEGG and GO analyses to predict the target genes based on the above results. GO analysis mainly analyzes genes and gene products in terms of molecular function, cellular composition, and biological process. By enrichment analysis of the three downregulated circRNAs in CeAD patients, the top 10 enrichment categories were selected for molecular function, cellular composition, and biological process (Figure 5A). The higher the level of GO enrichment, the greater the significance of the disease.

Functions in biological process included amyloid precursor protein metabolic process, positive regulation of focal adhesion assembly, and positive regulation of cell-substrate junction organization. The functions in cellular composition included hippocampal mossy fiber to CA3 synapse, presynapse, and presynaptic membrane. The functions in molecular function included deacetylase activity, histone deacetylase activity, protein deacetylase activity.

KEGG analysis was performed to study the signaling pathways between the target genes. The top five signaling pathways included Cushing syndrome, GABAergic synapse, relaxin signaling pathway, cortisol synthesis and secretion, and phospholipase D (Figure 5B).

#### 3.4.2. circRNA-miRNA-mRNA interaction network

**Figure 6:**
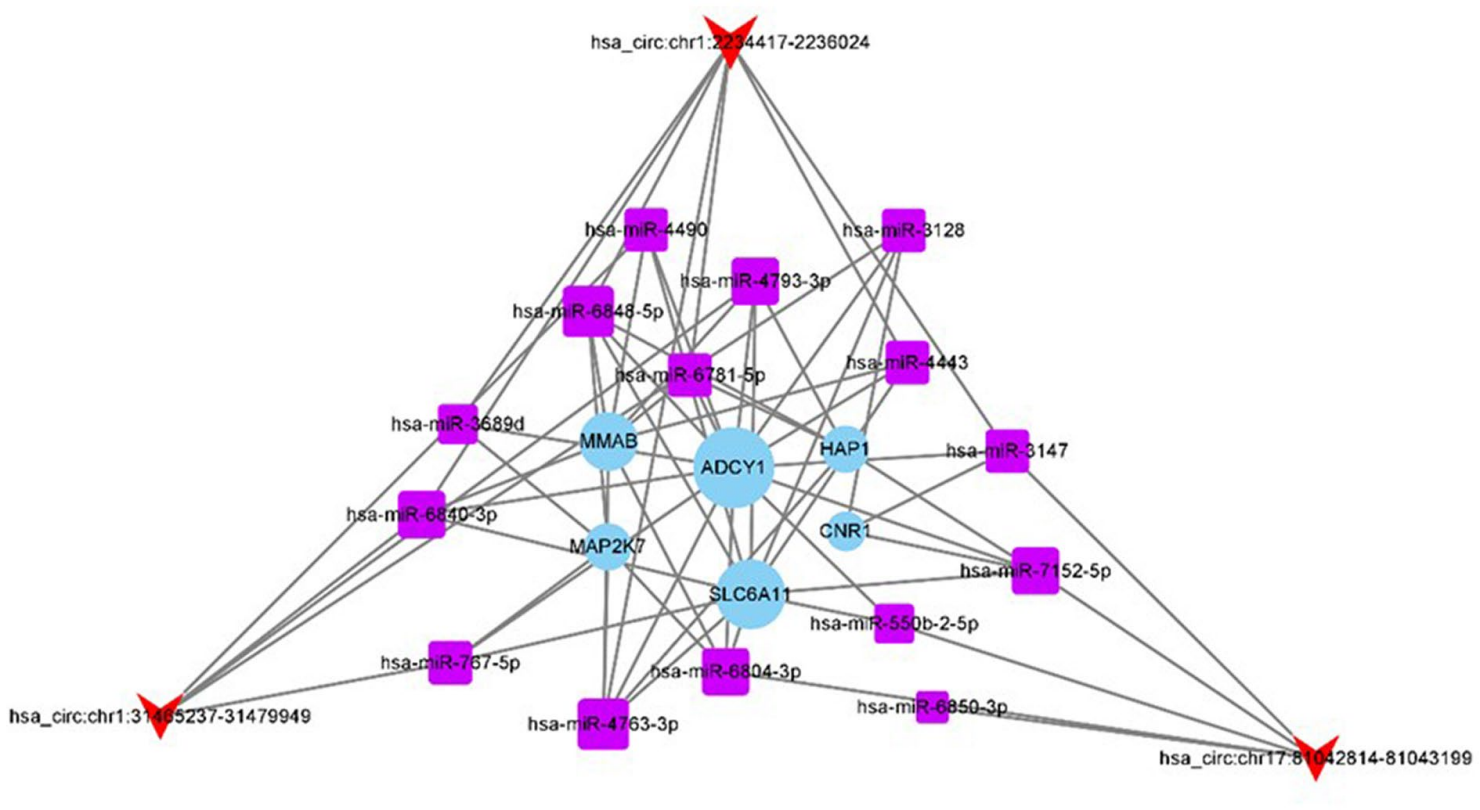
circRNA-miRNA-mRNA interaction network.

Owing to the sponge adsorption function of circRNAs, they can compete a miRNAs and affect the expression of target genes. To explore the effect of three differential circRNAs (has_circ:chr17:81042814–81,043,199, has_circ:chr1:2234417–2,236,024, and has_circ:chr1:31465237–31,479,949) on CeAD, we used the miRanda and TargetScan tools to predict miRNA binding sites on circRNAs. We predicted five miRNA binding sites with the highest match values for the three differentially expressed circRNAs. Subsequently, the miRanda and TargetScan tools were used for the target gene prediction of the 15 miRNAs, and a total of 208 target genes were obtained. After the KEGG pathway enrichment analysis, the top 10 pathways with the most significant enrichment results were screened for 26 target genes, and “total score” > 500 was set as the threshold for miRanda, and six target genes were predicted. Finally, we constructed a network diagram of 3 circRNAs, 15 miRNAs, and 6 target genes (Figure 6).

## 4. Discussion

This study is the first to analyze patients with CeAD at the circRNA level. By comparing the difference in circRNAs expression in patients with CeAD and healthy controls through high-throughput sequencing, we detected 460 circRNAs that were differentially expressed between the CeAD and control group, including 240 upregulated circRNAs and 220 downregulated circRNAs.

Studies on circRNAs have confirmed that they play an important role in ischemic stroke (14, 19). In particular, circRNAs are closely associated with risk factors for cerebrovascular diseases, such as atherosclerosis, hyperlipidemia, and diabetes. Endothelial damage is an important cause of atherosclerosis, and endothelial cells are damaged when the endothelium is stimulated by inflammatory factors, oxidative stress, and diabetes mellitus. It was confirmed that miR-197 can act as a target for has_circ_0068087, to mediate NLRP3 inflammatory vesicles and the NF-κB inflammatory pathway to enhance endothelial cell inflammation (18), as well as endothelial cell apoptosis, which has been implicated in atherosclerotic lesions. A study on hypoxic human umbilical vein endothelial cells have revealed that has_circ_0010729 acts as a sponge for miR-186 and promotes endothelial cell apoptosis by downregulating miR-186, suggesting that hsa_circ_0010729 plays an important role in affecting plaque stability (20). Atherosclerosis is the predominant cause of ischemic stroke, whereas CeAD is the most common cause of stroke in young people, both of which can lead to neurological impairment due to ischemia and hypoxia in the brain tissue. Given the findings on atherosclerosis in terms of circRNAs, we hypothesized that similar pathophysiological processes are involved in cerebral infarction due to cervical artery dissection. The occurrence of cervical artery dissection leads to narrowing or even occlusion of the arterial lumen, which triggers stroke events at the macro level and includes important processes, such as immunity and inflammatory responses at the micro level (21). In this study, circRNA-miRNA-mRNA networks were constructed based on the differentially expressed circRNAs, has_circ:chr17:81042814–81,043,199, has_circ:chr1:2234417–2,236,024, and has_circ:chr1:31465237–31,479,949. Our analysis revealed that has_circ:chr1:2234417–2,236,024 contained binding sites for miR-4443. This correlation suggests that miR-4443 may play an important role in ischemic stroke-induced immunosuppression. In ischemic stroke patients, miR-4443 interacts with the 3’-UTR of TRAF4 and inhibits TRAF4 protein expression. In addition, lipopolysaccharides and interleukin 4 can regulate miR-4443, and overexpression of miR-4443 also inhibits the TRAF4/IKA/NF-κB signaling pathway (19). Therefore, we believe that has_ circ: chr1:2234417–2,236,024 is involved in stroke events triggered by cerebral arterial dissection and plays a role in post-stroke immune processes.

To further predict the function of circRNAs in CeAD in this study, KEGG, and GO analyses were performed on differentially expressed circRNAs. GO analysis revealed that the amyloid precursor protein metabolic process, hippocampal mossy fiber to CA3 synapse, deacetylase activity, HSP protein, and other processes are related to protein metabolism, synapse, and regulation. Recent studies on HSP proteins have shown that both HSP27 and HSPB1 are highly expressed in patients with thoracic aortic coarctation and that their levels are closely related to CeAD prognosis (22).The pathogenesis of thoracic aortic coarctation is similar to that of cervical artery dissection, except for the parts of the disease; therefore, we speculate that HSP proteins may have a close relationship with arterial dissection.

The KEGG analysis of pathways related to the pathogenesis of CeAD mainly highlighted the phospholipase D signaling, ErbB signaling, relaxin signaling, and estrogen signaling pathways, apart from other pathways. The relaxin signaling pathway has been shown to inhibit inflammatory responses through multiple signaling pathways, which are associated with inflammation and apoptosis (23). A study suggested that estrogen has neuroprotective and anti-inflammatory effects (24). The pathophysiological process of ischemic stroke includes inflammation and immune response. Therefore, the above results suggest that has_ circ:chr17:81042814–81,043,199, has_circ:chr1:2234417–2,236,024, and has_circ:chr1:31465237–31,479,949 may play an important role in the progression of CeAD leading to stroke. CircRNAs are highly conserved and can produce sponge adsorption effect on miRNA, the function of downstream target genes is affected. Therefore, we believe that the differentially expressed circRNAs in CeAD patients can be used as a biomarker for the diagnosis of CeAD, which needs further verification in the future.

Cervical artery dissection is a common cause of stroke in young adults. This study find the changes in circRNAs in patients with CeAD. Based on the characteristics of circRNAs, we hope to make a breakthrough in the diagnosis and treatment of CeAD. Studies demonstrated that the regulation of circRNA through overexpression, gain or loss of function can affect diseases. The overexpression of specific circRNAs can be achieved using adenoviral or lentiviral vectors carrying circRNA sequences (25). Zolgensma, an FDA-approved adeno-associated virus (rAAV) gene therapy, has been used in diseases including hemophilia, Duchenne muscular dystrophy, and other diseases. Among them, it has achieved remarkable results in the treatment of patients with spinal muscular atrophy (26, 27). Similarly, the emergence of CRISPR-Cas9 technology also provides the possibility of gene editing therapy, but before circRNA is ready for treatment, it is also necessary to fully consider the limitations and adverse reactions, how to reduce off-target effects, improve the success rate of tissue targeting, and potential problems such as immunogenicity.

This study has certain limitations. First, the sample size is insufficient, and the number of patients studied needs to be increased to further verify the results of this study. Second, as an exploratory study, another limitation of this study is the lack of correction for multiple comparisons. Third limitation is that we cannot determine with certainty whether ischemic stroke or dissection will lead to changes in circulating RNA, as a non-dissecting stroke could also be a cause. We will establish a group of stroke caused by non-dissection causes in future experiments.

In summary, our study demonstrate differentially expressed CircRNAs in PBMCs of patients with CeAD for the first time. In view of the research achievements of CircRNAs in neurological diseases, we found that CircRNAs are involved in the pathophysiological process of CeAD pathogenesis through bioinformatics analysis of CircRNAs, which proposed a new direction for the diagnosis and treatment of CeAD.

## Data availability statement

The datasets presented in this study can be found in online repositories. The names of the repository/repositories and accession number(s) can be found in the article/supplementary material.

## Ethics statement

The studies involving humans were approved by Ethics Committee of the Shenzhen Hospital of Southern Medical University. The studies were conducted in accordance with the local legislation and institutional requirements. The participants provided their written informed consent to participate in this study. Written informed consent was obtained from the individual(s) for the publication of any potentially identifiable images or data included in this article.

## Author contributions

YLiu and LL designed the experiments. YW and ZD performed the experiments and collected the data. JL, YLi, JM, WT, and SY analyzed the data. YW wrote the manuscript. All authors contributed to the article and approved the submitted version.

## Footnotes

^1^ http://genome.ucsc.edu/
